# Barriers and Enablers to Implementing Teledentistry From the Perspective of Dental Health Care Professionals: Protocol for a Systematic Quantitative, Qualitative, and Mixed Studies Review

**DOI:** 10.2196/44218

**Published:** 2023-07-26

**Authors:** Pascaline Kengne Talla, Camille Inquimbert, Aimée Dawson, Diana Zidarov, Frédéric Bergeron, Fatiha Chandad

**Affiliations:** 1 Faculty of Dental Medicine and Oral Health Sciences, McGill University Montreal, QC Canada; 2 Faculty of Dental Medicine, University of Montpellier Montpellier France; 3 Faculty of Dental Medicine, Laval University Quebec City, QC Canada; 4 School of Rehabilitation, University of Montreal Centre Intégré Universitaire de la Santé et des Services sociaux du Centre Sud-de-l’île-de Montréal Centre de recherche interdisciplinaire en réadaptation du Montréal métropolitain Montreal, QC Canada

**Keywords:** teledentistry, oral health, barrier, enabler, dental health care providers, information and communication technology, protocol, dental, dentist, telehealth, telemedicine, theoretical domain framework, remote care, virtual care, perception, facilitator, systematic review, review method, librarian, PRISMA

## Abstract

**Background:**

There is growing literature on the potential of digital technologies for improving access to, ensuring continuity and quality of health care, and to strengthen health systems. Some studies have reported the cost-effectiveness of teledentistry, its reliability for remote dental screening, diagnosis, consultation, and treatment planning. Nonetheless, current evidence suggests that teledentistry implementation faces many challenges and is not yet adopted by dental health care providers (DHCPs). Developing strategies to improve teledentistry adoption requires an understanding of the factors that promote or hinder its successful implementation.

**Objective:**

This systematic review aims to identify and synthetize barriers and enablers to implementing teledentistry as perceived by DHCPs in their clinical practices, using the Theoretical Domains Framework (TDF) and the Capacity, Opportunity, and Motivation Behavior (COM-B) model.

**Methods:**

This protocol follows the PRISMA-P (Preferred Reporting Items for Systematic Reviews and Meta-Analyses Extension for Protocols) checklist. Literature will be searched in the following databases: PubMed, Cochrane Library, Web of Science, CINAHL, Embase, and PsycINFO. We will perform additional searches on Google, Google Scholar, and ProQuest Dissertations & Theses Global, screen the references of the included studies to capture additional relevant studies, and contact the authors of studies if we need more details. We will consider studies using qualitative, quantitative, and mixed methods. There will be no restrictions on the publication date and dental setting. We will include studies published in French, English, and Portuguese. Two independent reviewers will select the study, extract data, and assess methodological quality using the Mixed Methods Appraisal Tool’s checklist. Data analysis will include a descriptive and a thematic content analysis. We will synthetize and categorize the barriers and enablers using the TDF and COM-B model and present a narrative synthesis of our results using tables, figures, and quotes.

**Results:**

By March 2023, the literature search has retrieved 7355 publications. We will identify the range of barriers and enablers to implementing teledentistry through DHCPs’ perspectives. Considering the critical need for theory-based implementation interventions to improve the use of evidence-informed practices, we will synthesize the factors influencing the adoption of teledentistry based on the TDF domains and the 3 essential conditions predicting behavior change in accordance with the COM-B model. As needed, we will include additional determinants if not included in the TDF. We will conduct some subgroups analyses if studies are sufficient. We expect to complete the review by July 2024.

**Conclusions:**

This review will provide some insights on the determinants of teledentistry implementation as perceived by DHCPs in dental settings. These findings will cater to patients, families, DHCPs, researchers, academic and professional decision-makers, and policy makers. The results of the systematic review could be used to develop theory-led interventions in improving teledentistry implementation.

**Trial Registration:**

PROSPERO CRD42021293376; https://www.crd.york.ac.uk/prospero/display_record.php?RecordID=293376

**International Registered Report Identifier (IRRID):**

PRR1-10.2196/44218

## Introduction

Oral diseases significantly affect overall health, well-being, and quality of life and cause an increased economic burden at the patient and society levels. The Global Burden of Disease study in 2019 estimated that oral diseases affect close to 3.5 billion people across the world [1]. Despite interventions developed to improve oral health, numerous oral health inequalities persist over time and space with the greatest burden among marginalized people [1,2], resulting in poor oral health status. Considering the social and economic burdens related to oral health diseases and conditions, and their interconnection with some prevalent noncommunicable diseases [3], there is an international movement toward the imperative need to place oral health care within universal health coverage (UHC) and to improve essential oral health care [4], and to identify optimal strategies to improve oral health and oral health-related quality of life. Consequently, digital health tools are a promising means to address peoples’ health needs related to expanding UHC [5], in improving access to, continuity of, and quality of care and to strengthen health systems [6,7].

A growing body of evidence reports the potential of teledentistry, at the intersection of dentistry and telecommunications, as highly pertinent to the future of dentistry [8]. Teledentistry aims to use technologies and related services to deliver oral health care. It has proven to be effective and reliable for remote dental screening, diagnosis, consultation, and treatment planning [9,10]. Several reviews have reported the effectiveness and validity of teledentistry for prevention and health promotion and its accuracy for the diagnosis of oral diseases with results that are comparable to those of face-to-face interventions [10-13]. Dental health care providers (DHCPs), patients, and caregivers also have favorable attitudes and good satisfaction regarding digital interventions in dentistry [8]. Teledentistry has the potential to improve patients’ experiences [14,15], population health [16-18], DHCPs’ experiences [8,19] and to reduce oral care costs [20-22].

While valuable insights to the implementation of teledentistry can be obtained from decision-makers, patients, and caregivers, it is greatly recognized that DHCPs are among the key actors for the successful digital transformation [6,22,23]. As health professionals, DHCPs’ clinical acceptance are among the most important influences on the successful uptake, spread, and sustainability of teledentistry [23]. Consequently, DHCPs’ behaviors are an important proximal determinant of the quality of care provided to patients [24]. In their position as sources of information and opinion leaders who shape the clinical encounter, they could ensure teledentistry implementation and sustainability [25]. Their perspective is important to increase the expansion of teledentistry implementation and to deliver the right information to the right people at the right time. A better understanding of barriers and enablers that influence DHCPs’ behavior is a prerequisite to the successful implementation of teledentistry, as its adoption can help improve the patients’ outcomes and experiences.

Despite the promises of teledentistry, some empirical and theoretical studies have shown that teledentistry implementation remains a challenge [23,24,26,27]. Previous studies have reported that teledentistry has not yet been widely adopted by DHCPs [28-30]. The variation in the use of web-based oral health care cannot be explained by clinical factors or technical aspects alone. Structural, organizational, and DHCP- and patient-related factors including the absence of legal regulation of teledentistry, poor internet connection, lack of reimbursement, lack of training, and lack of familiarity with teledentistry [31,32] have been identified as barriers to implementing teledentistry. To maximize teledentistry implementation, it is crucial to understand DHCPs’ perspectives and to support them to modify their clinical behavior toward this modality of the delivery of oral health care. Understanding the barriers and enablers to the implementation of teledentistry is imperative to inform the design and the development of successful theory-led interventions, as well as the appropriate educational resources for the future implementation of teledentistry in dental practices.

Currently, there is no strong evidence available to inform decision-making, nor is there a tailored theoretically informed intervention designed to enhance its successful implementation. Systematic reviews offer a way to synthesize the broad range of barriers and enablers reported in the literature and to provide a broader, in-depth understanding of the factors influencing teledentistry implementation. Furthermore, many studies have reported the relevance of a theoretical framework to categorize the factors that influence health care professionals’ behaviors, inform the selection of behavior change techniques (BCTs) [24,33], and develop theory-driven and tailored strategies to support the effective implementation of any innovation [34-36]. To our knowledge, there has been no review of available evidence regarding DHCPs’ perspectives about the determinants of teledentistry implementation, nor has there been a previous systematic review using a theoretical framework to describe factors that may influence its implementation. This systematic review, therefore, aims to identify and synthesize barriers and enablers reported by DHCPs to implementing teledentistry in their clinical practices, using the theoretical domain framework and the Capacity, Opportunity, and Motivation Behavior (COM-B) model to determine factors predicting behavior change. This review protocol will address the question: “what are the barriers and enablers to teledentistry implementation that are reported by DHCPs in dental settings?”

Findings from this review will be valuable to inform the designing, planning, and implementation of teledentistry in any dental setting.

## Methods

### Study Design

The protocol for this review is registered on PROSPERO (CRD42021293376). This protocol has been written in accordance with the PRISMA-P (Preferred Reporting Items for Systematic Reviews and Meta-Analyses Extension for Protocols) checklist [37]. The systematic review will be reported in accordance with the PRISMA checklist [38].

### Eligibility Criteria

We will follow the PICOS (participants, interventions, comparators, outcomes, and settings) framework [39].

### Participants

We will include studies about the adoption of teledentistry by DHCPs. The DHCPs are dental professionals directly involved in clinical acts with patients. This review will include any dental professionals involved in oral health care. These DHCPs include general and specialist dentists, dental hygienists, dental therapists, dental nurses, dental students, dental hygiene students, and dental therapy students. All specialties in dentistry will be considered (eg, orthodontics, oral medicine, oral pathology, prosthodontics, periodontics, endodontics, pedodontics, and oral and maxillofacial surgery). If other participants such as patients, other health professionals, or managers are involved in a study with these DHCPs, the study will be included.

### Intervention

We will include only those studies that use teledentistry for the delivery of oral health care. Teledentistry interventions could be compared to usual or standard dental care or face-to-face dental visits (in-person) or no comparator. DHCPs can use several devices including cell phones, tablets, telephones, computers, personal digital assistants, text messaging, and apps [40-44] to interact with colleagues, other health professionals, or patients. We will include any studies on teledentistry delivered through different modalities such as synchronous (in real time; ie, direct interaction between a person [patient, caregiver, or health professional] and a DHCP) and asynchronous (store and forward; ie, transmission of health information to DHCPs to evaluate a patient’s condition or render a service outside of a real-time or live interaction) modalities. These interventions can be delivered through videoconference (eg, Zoom), telephone, websites, or any applications such as WhatsApp or other web-based mechanisms.

### Types of Studies to Be Included

We will include all studies with an original data collection. If there are several publications on the same project presenting similar data, we will include only the most recent publication, and if results are complementary and relevant, we will include all of them. We will include quantitative, qualitative, and mixed methods studies.

### Outcomes

We will include studies with different synonyms related to teledentistry implementation such as adoption, usage, uptake, readiness, and intent to its use. We will include studies reporting factors that could influence teledentistry implementation as reported by DHCPs; for instance, knowledge, intention, behavior, self-efficacy, satisfaction, skills, social influences, environmental context, and resources. We will include studies where data are collected using a variety of techniques, including individual interviews, focus groups, or questionnaires. We will include studies using validated or nonvalidated measurement tools to collect data. In this review, we will consider an enabler as any circumstance, factor, or variable that positively influences teledentistry implementation. A barrier will refer to any circumstance, factor, or variable preventing teledentistry implementation.

### Settings

We will consider all dental settings such as dental offices, community-based care, hospitals, schools, long-term care facilities, nursing homes, home dental care, university, and private or public dental settings. We will consider any geographical setting (eg, rural or urban regions) and any country (low-, middle-, or high-income).

### Exclusion Criteria

We will exclude any study using teledentistry only for education, training, and research. Studies may include other participants such as patients and other health care professionals, but DHCPs must be part of the studied population in the methodology, with the possibility to extract information from DHCPs. We will exclude studies that do not have a clear distinction between DHCPs and other participants’ findings. We will exclude any study focusing on health care providers other than DHCPs. We will exclude any studies focusing on the features of a platform for teledentistry, or the validity of teledentistry. Studies that are limited to describing the feasibility, usability, or technical evaluation of certain systems, interfaces or platforms will also be excluded. We will exclude editorials, letters, abstracts, reviews, commentary reports, legal issues, protocols, and press releases.

### Information Sources and Search Strategy

A health science information specialist will assist us in developing the literature search strategy in PubMed and will translate it after validation and revision in other electronic databases. A second health science information specialist will review the initial search strategy with the Peer Review of Electronic Search Strategies Tool [45]. The main concepts considered in the search strategy are “teledentistry,” “telemedicine oral health,” or “remote dental care,” “remote oral care,” “virtual dental care,” or “virtual oral care” and “dental health care providers,” “health personnel,” or “dentistry.” The electronic search will be carried out to identify all relevant studies from the following databases: PubMed, Cochrane Library, Web of Science, CINAHL, Embase, and PsycINFO. We will perform additional searches in Google, Google Scholar, and ProQuest Dissertations & Theses Global. We will screen the references of included studies to capture additional relevant studies.

There will be no restriction on the date of publication of the studies. We will include studies published in French, English, and Portuguese. A draft search strategy for MEDLINE has been developed with the support of a health science information specialist and is available in Multimedia Appendix 1.

### Selection Process and Data Collection Process

#### Data Management

We will import the list of generated references into EndNote X9 (Clarivate Analytics) software. We will identify and remove all duplicates. Retained studies will be uploaded in Covidence [46] for data screening and selection.

#### Selection Process

Two independent reviewers will select the retrieved studies. They will perform a calibration exercise with 10% of the total number of identified studies to ensure a mutual understanding of the eligibility criteria and to identify areas where further clarification is needed. Following the conclusive pilot test, the 2 reviewers will independently select studies at 2 levels: the first level of selection will be based on the titles and abstracts only (providing codes of “Yes,” “No,” or “Maybe”), and the second level will concern the full texts. All studies coded as “Yes” or “Maybe” will be further reviewed in full text by both reviewers. We will report the reasons for the exclusion of any retrieved or selected study.

Discrepancies will be resolved though discussion and consensus or with the consultation of a third reviewer. The selection stage will be considered an iterative process, allowing the eligibility criteria to be refined by the research team. We will contact authors for articles that cannot be obtained through institutional holdings.

#### Data Extraction

Two reviewers will extract the data independently using an Excel (Microsoft Corp) data extraction sheet. A calibration exercise will be independently performed by both reviewers on 5% of the included studies. The data extraction sheet will be modified and revised as necessary during the process of extracting data. Consistent with the abovementioned steps, data charting will be considered an iterative process as the review develops. Any disagreements that arise between the reviewers will be resolved through discussion and consensus, or with the consultation of a third reviewer. The draft extraction form is provided in Multimedia Appendix 2. The following data will be extracted:

- Characteristics of the included studies (eg, title, main author, year of publication, country of publication, language of publication, aims, funding, type of publication, study design, dental care settings, use of a theoretical framework, name of the theoretical framework, methods of data collection, limitations, and conclusions)
- Participant characteristics (eg, profile, sample size, sex, gender, age, general dentist or specialist, type of specialty, type of practice, and region of practice)
- Characteristics of teledentistry (eg, synchronous or asynchronous)
- Study outcomes (eg, knowledge, practices, beliefs, self-efficacy, time, compliance, behavior, teledentistry adoption and use, training, reimbursement, and equipment).

We will contact the authors if we need additional information on the study.

### Risk of Bias and Quality Assessment

Two independent reviewers will assess the methodological quality of the included studies using the core quality criteria in the Mixed Methods Appraisal Tool (MMAT) [47]. The MMAT is a reliable tool, and its use has been widely reported in the literature to facilitate the critical appraisal of mixed studies reviews [48-51]. It was first reported in 2009 as a single tool to evaluate the methodological quality of 5 categories of study designs: qualitative studies, randomized controlled trials, nonrandomized studies, quantitative descriptive studies, and mixed methods studies [50]. Further attempts to refine and improve the MMAT led to the development of its current version in 2018, based on the findings from a literature review on critical appraisal tools, pilot and interrater reliability testing, interviews with MMAT users, and the Delphi approach [52]. There are 5 questions each for quantitative and qualitative study designs and 15 for mixed method studies. The answer categories include “yes,” “no,” or “can’t tell” [47]. As required by Hong et al [47], we will present in detail the ratings of each criterion to better inform the quality of the included studies. We will present the overall quality score of the included studies using a descriptor such as an asterisk (*) or the percentage of quality criteria met [49]. For instance, 1 (*) refers to 20% of the quality criteria met, while 5 (*) refers to 100% of the quality criteria met. For mixed methods studies, we will provide 3 scores including one for qualitative, one for quantitative, and one for mixed methods components. The overall quality of the mixed methods study cannot exceed that of its weakest component [49].

We will consider for the included studies the following cutoff: studies with 1 and 2 (*) will be considered as being of low quality, 3 stars as medium quality, and 4 and 5 stars as high quality. Any discrepancy will be resolved through discussion and consensus or with the consultation of a third reviewer.

### Data Synthesis

A narrative synthesis will be conducted. We will analyze the data extracted using descriptive statistics (eg, frequencies and percentages). For instance, we will report the types of study designs, type of teledentistry interventions, countries where studies were conducted, and the study characteristics. We will synthesize and map the factors influencing teledentistry implementation in accordance with the theoretical domains of Theoretical Domains Framework (TDF) [53] and the context of information reported by the authors in order to allocate the factor to the appropriate theoretical domain. The likelihood is relatively low to miss a potential determinant of teledentistry implementation considering the wide range of factors considered within the TDF. We will report any new construct that is not considered in the TDF.

The robust theoretical model of TDF is more appropriate for this review because of its comprehensiveness. It is a promising and useful way of mapping the broad range of factors that influence a range of behaviors. It has been used in the literature to synthesize and classify the different determinants of a behavior and to target the relevant interventions [54,55]. In its original version, the TDF was composed of 12 theoretical domains (eg, knowledge, skills, social or professional role and identity, beliefs about capabilities, beliefs about consequences, motivation, goals, memory attention and decision processes, environmental context and resources, social influences, emotion, and action planning) that were derived from 33 behavior change theories using a process of expert consensus. The domains are considered as potential determinants of behavior and clearly indicated as barriers and enablers or factors positively or negatively influencing the behavior being studied. Some years later, the TDF has been refined and included in its revised version 14 theoretical domains and 84 theoretical constructs that can influence behavioral change [54]. These 14 individual and environmental theoretical domains include knowledge; social or professional role and identity; beliefs about capabilities; beliefs about consequences; skills; goals; optimism; reinforcement; intention; memory, attention, and decision processes; environmental context and resources; social influences; emotion; and behavioral regulation [54] (Multimedia Appendix 3).

Authors of previous reviews on teledentistry implementation have identified several potential factors influencing its implementation such as awareness, attitudes, knowledge, skills, training, lack of reimbursement, lack of infrastructure, self-confidence, organizational difficulty, quality of information, fear, lack of evidence, lack of guidelines, and data security issues [16,30,31,56]. These constructs can fit under the TDF domains of knowledge, skills, beliefs of capacities, beliefs about consequences, reinforcement, goals, emotion, identity and social roles or responsibilities, environmental context, and resources. In a systematic review of reviews on the barriers and facilitators to clinical behavior change by primary care practitioners, the authors have reported that 3 TDF domains were most commonly reported across many reviews, regardless of the type of behavior change and context: knowledge, environmental context and resources, and social influences [57].

Based on this information and our experience in dentistry, the preliminary step of selection of the TDF domains’ constructs could include without exhaustivity: procedural knowledge, knowledge, skills, skills assessment, competence, professional identity or role, self-efficacy, beliefs, attitudes, incentives or reimbursement, resources, infrastructures, social norms, fear, stress, and breaking habits.

The domains of the TDF are further mapped in 3 major essential conditions predicting behavior change, which are components of the COM-B model [33]. The COM-B component “Capability” can be psychological or physical and includes knowledge and skills. The component “Opportunity” can be social or physical and could include social influences and environmental context or resources. Finally, the component “Motivation” can be a reflective process, or automatic process and could include intention and emotions. This approach guides the understanding of behavior in all contexts. The COM-B model is a part of the “behavior system,” which helps to design interventions [33] through BCTs [58]. According to the analysis of the nature of behavior and the relevant mechanisms to elicit change, the Behavior Change Wheel is a reliable framework that aims to link the determinants of the target behavior to the design and selection of interventions and policies (Multimedia Appendix 4). Considering the critical need for theory-based implementation interventions to improve the use of evidence-informed practices, we will organize the barriers and enablers by these essential conditions, which could be behavioral change components with high potential to improve teledentistry implementation. If possible, we will perform subgroup analysis based on the number of eligible studies with the following variables: age, gender, type of dental professionals (eg, dentists versus dental hygienists), category of dentists (eg, generalist and specialists), type of setting (eg, private or public), and time period (before and after 2020 with the COVID-19 pandemic), and their methodological quality. Results will be reported in tabular form, graphics, and quotes to reflect the extent of included literature.

At the end of the review, we will have an in-depth understanding of barriers and enablers from DHCPs’ perspectives as well as the comprehensiveness of TDF domains, constructs, and predicting factors.

## Results

As of March 2023, we have updated the search strategy. The search strategy identified 7355 studies. This systematic review will aim to identify and synthetize the barriers and enablers to teledentistry implementation from DHCPs’ perspectives. We will present the sociodemographic information or moderators reported by the authors of these studies, which could influence behavior change. Furthermore, possible research gaps may be identified to guide future studies. Our results will be reported in accordance with the PRISMA-P checklist [38]. We expect to complete the review and publish the results within a year of publication of this protocol—around July 2024.

## Discussion

### Anticipated Findings

This review will identify and synthesize the enablers and barriers influencing teledentistry implementation from DHCPs’ perspectives. These factors can represent micro to macro levels of analysis, which will affect implementation success and predict implementation efforts. The TDF, one of the robust and common theoretical frameworks used in implementation science, will allow us to comprehensively classify the range of barriers and enablers related to teledentistry implementation, which could assist later in the development of tailored implementation interventions [35]. The TDF has also been used as a coding framework for the analysis of barriers in systematic reviews [34,59,60]. However, to our knowledge, its use is limited in dentistry and was not used to synthetize the factors influencing teledentistry implementation as reported by DHCPs. The knowledge of determinants from the TDF and COM-B model will inform the selection of targeted BCTs, which, in turn, can be used to design behavior change interventions to improve the implementation of teledentistry. We will present the strengths and limitations of this review. As such, implications of the review findings for future research, practice, and policy will be discussed and presented.

### Conclusions

This systematic review protocol presents the methodological steps aiming to identify and synthetize the barriers and enablers to implementing teledentistry from DHCPs’ perspectives using strong theoretical frameworks. The results of this review can be relevant for different knowledge users including patients, DHCPs, researchers, managers, academics, policy makers, and professional decision-makers in regulatory bodies to design behavioral change interventions to improve the success of teledentistry implementation in dental settings.

## Appendix

Multimedia Appendix 1 Search strategy.

Multimedia Appendix 2 Data extraction sheet.

Multimedia Appendix 3 Definition of theoretical domains.

Multimedia Appendix 4 Match between TDF and COM-B.

## Abbreviations

BCT: Behavior Change Technique
COM-B: Capacity, Opportunity, and Motivation Behavior
DHCP: Dental health care providers
MMAT: Mixed Methods Appraisal Tool
PICOS: Participants, Interventions, Comparators, Outcomes, and Settings
PRISMA-P: Preferred Reporting Items for Systematic Reviews and Meta-Analyses Extension for Protocols
TDF: Theoretical Domains Framework
UHC: Universal Health Coverage

## Data Availability

The data sets generated and analyzed during this study will be available upon request to the corresponding author after publication of the review.

## Amendments

We will publish any changes to this protocol as amendments on our PROSPERO protocol web page. We will declare any changes and the date of the amendments, including the rationale for them.
