# Supplementary material for: Barriers and Enablers to Implementing Teledentistry From the Perspective of Dental Health Care Professionals: Protocol for a Systematic Quantitative, Qualitative, and Mixed Studies Review
Source: JMIR Res Protoc. 2023 Jul 26;12:e44218. doi: 10.2196/44218 (PMC10413248; doi:10.2196/44218)
Supplement: Multimedia Appendix 1 [file resprot_v12i1e44218_app1.docx]

**Appendix 1: MEDLINE search strategy**

**Date of the first search:** 02-11-2022

| **#** | **Search strategy** | **Results** |
| --- | --- | --- |
| 1 | "teledentistry"[Title/Abstract] OR "tele-dentistry"[Title/Abstract] |  |
| 2 | telemedicine[Title/Abstract] OR tele-medicine[Title/Abstract] OR "remote screening"[Title/Abstract] OR teleconsultation*[Title/Abstract] OR tele-consultation*[Title/Abstract] OR telediagnosis[Title/Abstract] OR tele-diagnosis[Title/Abstract] OR "tele-health"[Title/Abstract] OR telehealth[Title/Abstract] OR telemonitoring[Title/Abstract] OR tele-monitoring[Title/Abstract] |  |
| 3 | "Telemedicine"[Mesh] |  |
| 4 | "mobile phone"[Title/Abstract] OR smartphone*[Title/Abstract] OR "mobile application*"[Title/Abstract] OR "mobile app"[Title/Abstract] OR "mobile apps"[Title/Abstract] OR "cellular phone"[Title/Abstract] OR "mobile phone"[Title/Abstract] OR "text messag*"[Title/Abstract] |  |
| 5 | "Cell Phone"[Mesh] OR "Mobile Applications"[Mesh] |  |
| 6 | #2 OR #3 OR #4 OR #5 |  |
| 7 | "Stomatognathic Diseases"[Mesh] OR "Dentistry"[Mesh] OR "Oral Health"[Mesh] |  |
| 8 | dental[Title/Abstract] OR dentist*[Title/Abstract] OR "oral surger*"[Title/Abstract] OR "maxillofacial surger*"[Title/Abstract] OR "oral cancer"[Title/Abstract] OR endodontic*[Title/Abstract] OR endodont*[Title/Abstract] OR orthodonti*[Title/Abstract] OR periodont*[Title/Abstract] OR prosthodont* [Title/Abstract] OR odontolog*[Title/Abstract] OR apicoectom*[Title/Abstract] OR gingivectom*[Title/Abstract] OR gingivoplast*[Title/Abstract] OR glossectom*[Title/Abstract] OR "mandibular advancement"[Title/Abstract] OR alveolectom*[Title/Abstract] OR alveoloplast*[Title/Abstract] OR vestibuloplast*[Title/Abstract] OR "root canal"[Title/Abstract] OR oral[Title/Abstract] OR oropharyng*[Title/Abstract] OR temporomandibular[Title/Abstract] OR TMJ[Title/Abstract] OR jaw[Title/Abstract] OR jaws[Title/Abstract] OR mandibular[Title/Abstract] OR maxillofacial[Title/Abstract] OR mandible*[Title/Abstract] OR maxilla*[Title/Abstract] OR "alveolar ridge"[Title/Abstract] OR orthognathic[Title/Abstract] OR tooth[Title/Abstract] OR teeth[Title/Abstract] OR occlusion[Title/Abstract] OR malocclusion[Title/Abstract] OR mal-occlusion[Title/Abstract] OR tongue*[Title/Abstract] OR glossal[Title/Abstract] OR buccal[Title/Abstract] OR palatal[Title/Abstract] OR palate[Title/Abstract] OR palates[Title/Abstract] OR labial[Title/Abstract] OR lip[Title/Abstract] OR lips[Title/Abstract] OR gingiva*[Title/Abstract] OR gingiviti*[Title/Abstract] OR "Oral Health"[Title/Abstract] |  |
| 9 | #7 OR #8 |  |
| 10 | #6 AND #9 |  |
| 11 | #10 OR #1 | 2539 |
