# Supplementary material for: Barriers and Enablers to Implementing Teledentistry From the Perspective of Dental Health Care Professionals: Protocol for a Systematic Quantitative, Qualitative, and Mixed Studies Review
Source: JMIR Res Protoc. 2023 Jul 26;12:e44218. doi: 10.2196/44218 (PMC10413248; doi:10.2196/44218)
Supplement: Multimedia Appendix 2 [file resprot_v12i1e44218_app2.docx]

**Appendix 2: Barriers and Enablers to Implementing Teledentistry From the Perspective of Dental Health Care Professionals: Protocol for a Systematic Quantitative, Qualitative, and Mixed Studies Review**

| **Title of the review:** Barriers and enablers to implementing teledentistry with dental healthcare professionals: Protocol for a systematic quantitative, qualitative, and mixed studies review | |
| --- | --- |
| **Reviewer’s name** |  |
| **Publication details** | Title |
|  | Year of publication |
|  | First author |
|  | Corresponding author |
|  | Author contact |
|  | Country in which the study is conducted |
|  | Socio demographics of country (developed, developing including low and middle income countries) |
|  | Language of publication |
|  |  |
| **Study details** | Objectives |
|  | Inclusion criteria |
|  | Exclusion criteria |
|  | Intervention |
|  | Comparators |
|  |  |
| **Methodological information** | Type of study (Quantitative, qualitative or mixed) |
|  | Theoretical approach (Y, N, NR) |
|  | Name of theoretical approach if yes |
|  | Study design (cross sectional, phenomenology, ethnography, grounded theory, case report, case control, cohort, economic evaluation) |
|  | Care setting (e.g. hospital, dental school/college or university, prison, dental office, community, CHLSD or nursing home or elders facilities), |
|  | Method of data collection (e.g. survey, individual interview, observation, focus groups) |
|  |  |
| **Participant information** | Participant profile (DHCP) (ex. dentist generalist, dentist specialist, dental hygienist, dental therapist, dental nurse, dental student, dental hygienist student, dental nurse student, dental specialist student or other/precise) |
|  | Type of speciality if reported: e.g. orthodontics, periodontics, prosthodontics, endodontics, pediatrics, OMFS/Oral medicine or other) |
|  | Age (mean, SD) of the dental health care providers |
|  | Gender |
|  | Participants sex (% female) |
|  | Type of practice (solo or group) |
|  | Total participants contacted |
|  | Total participants participated |
|  | Rate of participation if reported |
|  | Total participants included in study during the data analysis |
|  | Other participants |
|  |  |
| **Characteristics of teledentistry** | Types of teledentistry modalities e.g. synchronous, asynchronous, remote monitoring, mhealth) |
|  | Technology used e.g. adobe connect, skype, whatsapp, mobile phone, email, m phone, google meet, facebook |
|  |  |
| **Findings** | Primary outcomes |
|  | Secondary outcomes |
|  | Type of data analysis |
|  | Main findings |
|  | Limitations |
|  | Funding |
|  | Conflict of interest |
|  | Areas for future research |
