# Supplementary material for: Barriers and Enablers to Implementing Teledentistry From the Perspective of Dental Health Care Professionals: Protocol for a Systematic Quantitative, Qualitative, and Mixed Studies Review
Source: JMIR Res Protoc. 2023 Jul 26;12:e44218. doi: 10.2196/44218 (PMC10413248; doi:10.2196/44218)
Supplement: Multimedia Appendix 3 [file resprot_v12i1e44218_app3.docx]

**DEFINITION OF DOMAINS IN THEORETICAL DOMAINS FRAMEWORK**

| **Domains** | **Definition** | **Constructs (some examples)** |
| --- | --- | --- |
| Knowledge | An awareness of the existence of something | Scientific rationale or knowledge of the condition; knowledge of task environment |
| Skills | An ability or proficiency acquired through practice | Skills development; competence; ability |
| Social/professional role and identity | A coherent set of behaviours and displayed personal qualities of an individual in a social or work setting | Social identity; professional boundaries |
| Beliefs about consequences | Acceptance of the truth, reality, or validity about outcomes of a behaviour in a given situation | Beliefs; anticipated regret; outcome expectancies |
| Beliefs about capacities | Acceptance of the truth, reality, or validity about an ability, talent, or facility that a person can put to constructive use | Perceived competence; self-efficacy; self-esteem or confidence |
| Reinforcement | Increasing the probability of a response by arranging a dependent relationship, or contingency, between the response and a given stimulus | Incentives; Punishment; sanctions |
| Optimism | The confidence that things will happen for the best or that desired goals will be attained | Pessimism; optimism; identity |
| Social influences | Those interpersonal processes that can cause individuals to change their thoughts, feelings, or behaviours | Social norms; social support; group identity; modelling |
| Emotion | A complex reaction pattern, involving experiential, behavioural, and physiological elements, by which the individual attempts to deal with a personally significant matter or event | Affect; fear; anxiety; affect |
| Goals | Mental representations of outcomes or end states that an individual wants to achieve | Goal priority; target setting |
| Intentions | A conscious decision to perform a behaviour or a resolve to act in a certain way | Stages of change model |
| Environmental context and resources | Any circumstance of a person's situation or environment that discourages or encourages the development of skills and abilities, independence, social competence, and adaptive behaviour | Resources; materials; organisational culture or climate; social pressure |
| Memory, attention and decision making | The ability to retain information, focus selectively on aspects of the environment and choose between two or more alternatives | Attention; decision-making |
| Behavioral regulation | Anything aimed at managing or changing objectively observed or measured actions | Action planning; breaking habit |

[53]. Cane J, O’Connor D, Michie S. Validation of the theoretical domains framework for use in behaviour change and implementation research. Implement Sci 2012;7(1):37. doi: 10.1186/1748-5908-7-37
