# Supplementary material for: Barriers and Enablers to Implementing Teledentistry From the Perspective of Dental Health Care Professionals: Protocol for a Systematic Quantitative, Qualitative, and Mixed Studies Review
Source: JMIR Res Protoc. 2023 Jul 26;12:e44218. doi: 10.2196/44218 (PMC10413248; doi:10.2196/44218)
Supplement: Multimedia Appendix 4 [file resprot_v12i1e44218_app4.docx]

**The COM-B system - a framework for understanding behavior**

Physical e.g. skills

Psychosocial e.g. behavioral regulation; knowledge

Social e.g. social influences

Physical e.g. environmental context and resources

Reflexive e.g. goals; intention; beliefs about capacities

Motivation

Opportunity

Capacity

Implementation of teledentistry

Automatic e.g. optimism; emotion; reinforcement

**The Behaviour Change Wheel**


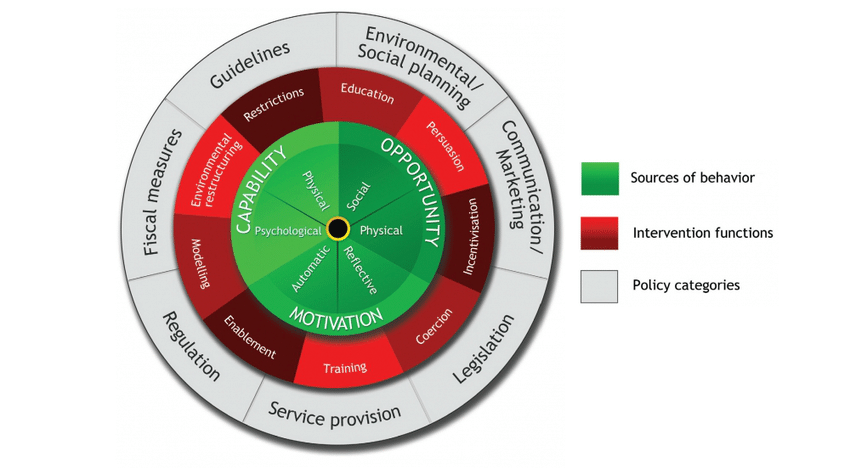


[30]. Michie, S., van Stralen, M.M. & West, R. The behaviour change wheel: A new method for characterising and designing behaviour change interventions. *Implementation Sci* **6**, 42 (2011)
